# Novel Compound Inhibitors of HIV-1_NL4-3_ Vpu

**DOI:** 10.3390/v14040817

**Published:** 2022-04-15

**Authors:** Carolyn A. Robinson, Terri D. Lyddon, Hwi Min Gil, David T. Evans, Yury V. Kuzmichev, Jonathan Richard, Andrés Finzi, Sarah Welbourn, Lynn Rasmussen, N. Miranda Nebane, Vandana V. Gupta, Sam Ananthan, Zhaohui Cai, Elizabeth R. Wonderlich, Corinne E. Augelli-Szafran, Robert Bostwick, Roger G. Ptak, Susan M. Schader, Marc C. Johnson

**Affiliations:** 1 Department of Molecular Microbiology and Immunology, University of Missouri, School of Medicine and the Christopher S. Bond Life Sciences Center, Columbia, MO 65211, USA; carolyn.robinson127@gmail.com (C.A.R.); lyddontd@missouri.edu (T.D.L.); sarahwelbourn@gmail.com (S.W.); 2 Department of Pathology and Laboratory Medicine, University of Wisconsin-Madison, Madison, WI 53705, USA; hwi.m.gil@vanderbilt.edu (H.M.G.); dtevans2@wisc.edu (D.T.E.); 3 Infectious Disease Research, Drug Development Division, Southern Research, Frederick, MD 21701, USA; yuryvk@gmail.com (Y.V.K.); zhcai2008@gmail.com (Z.C.); erwond@gmail.com (E.R.W.); rptak@southernresearch.org (R.G.P.); susanmschader@gmail.com (S.M.S.); 4 Centre de Recherche du CHUM, Montréal, QC HX2 0A9, Canada; jonathan.richard.1@umontreal.ca (J.R.); andres.finzi@umontreal.ca (A.F.); 5 Département de Microbiologie, Infectiologie et Immunologie, Université de Montréal, Montréal, QC HX2 0A9, Canada; 6 High-Throughput Screening Center, Drug Discovery Division, Southern Research, Birmingham, AL 35205, USA; lrasmussen@southernresearch.org (L.R.); mnebane@southernresearch.org (N.M.N.); bbostwick@southernresearch.org (R.B.); 7 Department of Chemistry, Drug Discovery Division, Southern Research, Birmingham, AL 35205, USA; vgupta@southernresearch.org (V.V.G.); sananthan@southernresearch.org (S.A.); caugelli-szafran@southernresearch.org (C.E.A.-S.)

**Keywords:** HIV, MLV, GaLV, inhibitors, HTS

## Abstract

HIV-1 Vpu targets the host cell proteins CD4 and BST-2/Tetherin for degradation, ultimately resulting in enhanced virus spread and host immune evasion. The discovery and characterization of small molecules that antagonize Vpu would further elucidate the contribution of Vpu to pathogenesis and lay the foundation for the study of a new class of novel HIV-1 therapeutics. To identify novel compounds that block Vpu activity, we have developed a cell-based ‘gain of function’ assay that produces a positive signal in response to Vpu inhibition. To develop this assay, we took advantage of the viral glycoprotein, GaLV Env. In the presence of Vpu, GaLV Env is not incorporated into viral particles, resulting in non-infectious virions. Vpu inhibition restores infectious particle production. Using this assay, a high throughput screen of >650,000 compounds was performed to identify inhibitors that block the biological activity of Vpu. From this screen, we identified several positive hits but focused on two compounds from one structural family, SRI-41897 and SRI-42371. We developed independent counter-screens for off target interactions of the compounds and found no off target interactions. Additionally, these compounds block Vpu-mediated modulation of CD4, BST-2/Tetherin and antibody dependent cell-mediated toxicity (ADCC). Unfortunately, both SRI-41897 and SRI-42371 were shown to be specific to the N-terminal region of NL4-3 Vpu and did not function against other, more clinically relevant, strains of Vpu; however, this assay may be slightly modified to include more significant Vpu strains in the future.

## 1. Introduction

Since the beginning of the HIV epidemic, more than 75 million people have been infected with HIV and about 33 million people have died as a result. While there have been advances in treating HIV, approximately one million people per year die from complications resulting from HIV infection, and about 38 million people are still living with HIV/AIDS (American Foundation for Aids Research, 2020). Importantly, HIV can remain infectious at low levels in a host, despite modern advances in combinational antiretroviral therapies (ART) that target HIV proteins such as integrase, reverse transcriptase, and protease. Furthermore, current therapies require life-long, daily treatment, which is expensive and unattainable for many of those living with HIV. Importantly, if drug treatment ceases, viral loads increase rapidly, resulting in a restoration of pre-treatment viral load levels that will continue HIV spread and eventually lead towards the development of AIDS [1,2,3,4,5]. Research efforts to further the understanding and control HIV are of utmost importance, as eradication of HIV is unlikely without a cure.

A potential target of future therapies is Vpu. Vpu is an HIV-1 accessory protein involved in viral assembly but not absolutely required for viral replication in vitro (reviewed in [6]). Vpu is between 81–86 amino acids long and has been well characterized in its role of downmodulating CD4 to prevent superinfection and antagonizing BST-2/Tetherin to facilitate viral release [7,8,9]. Additionally, Vpu has been reported to have other cellular targets including natural killer, T and B cell antigen (NTB-A) [10], CD155 (also known as polio virus receptor (PVR)) [11], CCR7 [12], CD62L and SNAT1 [13], although these targets are less characterized.

Vpu contains a transmembrane domain (TMD) at its N-terminus and a cytoplasmic tail domain (CTD) at its C-terminus. Much of the activity of Vpu has been shown to be dependent on the phosphorylation of two serine residues in the cytoplasmic tail (S52/56) that are phosphorylated by casein kinase-2 (CK-2) and used to interact with a cellular ubiquitin ligase [14,15]. Specifically, Vpu depends on the phosphorylation of two serine residues for its ability to interact with a member of the Skp1-cullin1-F-box complex (SCF) containing the specific F-box proteins βTrCP-1 or βTrCP-2 and connect to the proteasome degradation pathway [15]. Our lab has previously shown that in a cell line containing a CRISPR/Cas9 double knockout of the βTRCP proteins (1 and 2), Vpu activity against CD4 was abolished, and anti-BST-2/Tetherin activity was reduced significantly [16]. This suggests that Vpu has at least one additional mechanism used for counteracting BST-2/Tetherin. The S52/56 mutations also no longer affected Vpu activity against BST-2/Tetherin in the knockout cell line. This is consistent with previous findings that Vpu and BST-2/Tetherin interact using their transmembrane domains and that S52/56 function to recruit βTrCP [17,18].

The transmembrane domain of Vpu has been shown to be required for the antagonism of BST-2/Tetherin, specifically residues A14/18 [19]. Additionally, several poorly characterized targets of Vpu have been shown to be targeted by the transmembrane domain (A14/18 dependent) rather than the cytoplasmic domain, including NTB-A, PVR, and CD62L [10,11,20,21]. Targets of the transmembrane domain are generally thought to be downmodulated through mis-trafficking rather than degradation; however, this has yet to be fully elucidated [18]. Kueck, et al., 2015, suggested that Vpu interacts with AP1/AP2 Clathrin adapters to facilitate the endosomal degradation of BST-2/Tetherin in a manner that is also dependent on the S52/56 phosphoserines.

In addition to its well characterized role in targeting cellular proteins, Vpu has been shown to function as an ion channel for monovalent cations such as sodium and potassium [22,23]. While this function appears to be conserved, it is poorly understood and has yet to be linked to pathogenicity of HIV-1.

While most HIV-1 infected patients do not produce enough neutralizing antibodies to control the infection, the immune system can mediate the elimination of HIV-1 infected cells through antibody dependent cellular cytotoxicity (ADCC). During an ADCC response, antibodies bind to a foreign antigen on the surface of infected cells and recruit effector cells, including natural killer (NK) cells, that lyse the infected cell, eliminating virus-producing cells. In the case of HIV-1, the antigens on the surface of an infected cell are the envelope glycoproteins (Env). Several groups have shown that Vpu is an important player in protecting HIV-1 infected cells from lysis by ADCC [24,25]. CD4 downregulation prevents Env-CD4 interaction at the surface of infected cells, thus limiting the exposure of CD4-induced Env epitopes targeted by ADCC-mediating, non-neutralizing antibodies naturally present in the plasma from HIV-1-infected individuals [26,27]. Downregulation of BST-2/Tetherin, which otherwise traps viral particles at the surface of infected cells, contributes to limit the overall amount of Env on the surface of infected cells and to a lower ADCC response [27,28,29].

The impact of Vpu on viral spread suggests considerable therapeutic potential for a Vpu-specific inhibitor [30,31]. There have been several attempts to specifically target Vpu; however, many of the proposed compounds target the ion-channel activity of Vpu and have been shown to have a low selectivity index or non-Vpu-specific ion-channel targets [23,32,33]. Notably, the compound BIT225 is currently in Phase II of the Australian New Zealand clinical trials and has been shown to increase plasma derived activated CD4^+^ and CD8^+^ T cells and NK cells if coupled with ART when compared with patients being treated with ART alone [34]. However, it is important to note that BIT225 is not Vpu specific [33,35] and does not appear to affect the Vpu-mediated antagonism of BST-2/Tetherin [36]. There have been additional efforts to target the ubiquitin ligase machinery upstream of Vpu, such as with the compound MLN4924, but this compound does not rescue ADCC activity at tolerated levels in cells [37].

Finally, our lab has previously demonstrated that Vpu downmodulates Gibbon Ape Leukemia Virus Envelope (GaLV Env) through the targeting of the cytoplasmic tail region using the same SCF/βTrCP ubiquitin ligase [16,38,39]. Using this observation, we developed a novel assay for a gain-of-function high throughput screen for inhibitors of Vpu. Here, we identify a family of novel inhibitors specific to NL4-3 HIV-1Vpu. Two of the identified compounds, SRI-41897 and SRI-42371, were examined further and found to effectively block the Vpu downmodulation of GaLV Env, BST-2/Tetherin, CD4, and rescue ADCC killing of infected cells.

## 2. Materials and Methods

### 2.1. Plasmids

NL4-3 derived HIV-1-CMV-GFP was provided by Vineet Kewal Rammani (National Cancer Institute (NCI)—Frederick) and previously described [40]. This proviral vector lacks the accessory genes vif, vpr, nef, and env and contains a CMV promoter driven GFP in the place of nef. Both Vpu positive and Vpu negative constructs were used and described previously [38]. The infectious molecular clone NL4-3 was obtained through the NIH HIV Reagent Program, Division of AIDS, NIAID, NIH, contributed by M.A. Martin [41].

HIV-1 Gag-mRFP constructs were created in a two-step cloning process. First, the Nhe1- Xho1 region of NL4-3 derived HIV-1-CMV-GFP constructs (+/− Vpu) containing GFP were replaced with a Blasticidin Resistance gene gblock fragment ordered from Integrated DNA Technologies (IDT). Secondly, the BamH1-Xho1 region of a previously described Gag-mRFP construct gifted by Akira Ono (University of Michigan) was replaced with the BamH1-Xho1 region of the new constructs where Gag-mRFP replaces Pol [42].

The MLV/GaLV chimera construct used in infectivity assays was described previously [16,38,39]. The APOBEC3G-GFP was gifted by Daniel Salamango and described previously [43]. Plasmids for creation of stable cell lines were created by insertion of CDC25-GFP or MLV/GaLV into the retroviral transfer vector pQCXIH (Clonetech, Mountain View, CA, USA). The SHIV_KU-1vMC33_ strain of Vpu was gifted from Edward Stephens (University of Kansas), and the CH77 strain Vpu was inserted using a GBlock (IDT). Both Vpu strains were cloned into the NL4-3 derived HIV-1-CMV-GFP plasmid described above. The proline insertion mutant was created using PCR mutagenesis of NL4-3 Vpu and the subsequent infusion into the NL4-3 derived HIV-1-CMV-GFP plasmid described above.

### 2.2. Compounds

Compound SRI-40244 (T6143675, CAS # 1090371-79-1) and Compound SRI-41897 (Z285895332, CAS # 1208824-23-0) were purchased from Enamine. Compound SRI-42371 was purchased from Ambinter (Amb11288305 CAS # 2185475-57-2). Compounds were resuspended at 40 mM (stock concentration) in DMSO and stored at 4 °C.

### 2.3. Cell Lines

The base HEK293FT (293FT) cell line used for stable expression was originally obtained from Invitrogen (Carlsbad, CA, USA). For the GaLV inhibition assay, cell lines stably expressing GaLV/MLV chimera construct were transduced with NL4-3 derived HIV-1-CMV-GFP virus pseudotyped with VSV-G.

Counter-screen stable cell lines (AOBEC3G-GFP and CDC25α-GFP) were cloned into a pQCXIH retroviral transfer vector packaged and transduced into HEK293FT cells using VSV-G gylocprotein, then selected with hygromycin. In both cases, a single cell isolate was selected and propagated for use.

The 293T mCAT-1 cell line expressing the ecotropic F-MLV Env receptor was provided by Walther Mothes.

TZM-GFP cells were gifted by Massimo Pizzato and previously described [44].

All 293FT based cell lines and TZM-GFP cells were maintained in Dulbecco’s Modified Eagle Medium (DMEM) supplemented with 7.5% fetal bovine serum, 2 mM L-glutamine, 1 mM sodium pyruvate, and 10 mM nonessential amino acids.

The cell lines for the ADCC assay were obtained and cared for as previously described [45]. The NK cell line (KHYG-1 cells) was obtained from the Japan Health Sciences Foundation and transduced with the V158 variant of human CD16. The target cells (CEM.NKR-CCR5) were obtained from the AIDS Reagent Program and were modified as described by transducing with a vector carrying an SIV LTR driven luciferase reporter gene.

Primary human peripheral blood mononuclear cells (PBMCs) and CD4^+^ T cells were isolated, activated, and cultured as previously described [46]. Briefly, PBMCs were obtained by leukapheresis and CD4^+^ T lymphocytes were purified from resting PBMCs by negative selection using immunomagnetic beads per the instructions of the manufacturer (StemCell Technologies, Vancouver, BC, Canada) and were activated with phytohemagglutinin-L (10 μg/mL) for 48 h and then maintained in RPMI 1640 complete medium supplemented with recombinant interleukin-2 (rIL-2) (100 U/mL). VSV-G-pseudotyped HIV-1 NL4-3 virus was produced and titrated. Viruses were then used to infect activated primary CD4 T cells from healthy HIV-1-negative donors by spin infection at 800× *g* for 1 h in 96-well plates at 25 °C. Ethics Statement: Written informed consent was obtained from all study participants and research adhered to the ethical guidelines of CRCHUM and was reviewed and approved by the CRCHUM institutional review board (ethics committee, approval number CE16.164-CA). Research adhered to the standards indicated by the Declaration of Helsinki. All participants were adults and provided informed written consent prior to enrolment in accordance with Institutional Review Board approval.

### 2.4. GaLV Assay/Screen/Flow

Infectivity (GaLV) Assay. Cell lines stably expressing GaLV/MLV chimera construct were transduced with an aliquot of NL4-3 derived HIV-1-CMV-GFP virus pseudotyped with Vesicular Stomatitis Virus protein G (VSV-G) in a 10 cm cell culture dish. 24 h post-transduction, cells were separated into 6 or 12 well plates, and treated with compound DMSO (Sigma-Aldrich Cat# D4540), or MLN4924 (Calbiochem Cat# 5.05477.0001) for 24 additional hours at 37 °C. Viral media was collected and frozen at −80 °C for between 2 and 24 h. Once thawed, the virus was spun at 3000 RCF for 5 min to clear any debris. Viral supernatant (NL4-3 derived HIV-1-CMV-GFP pseudotyped with GaLV ENV/MLV chimera) was then used to transduce mCAT-1 cells for 48 h at 37 °C and collected for flow cytometry. GFP positive mCAT-1 Cells (indicating a successful transduction) were measured.

### 2.5. High Throughput Screen

Compound collection: Southern Research maintains a collection of 759,059 unique, non-proprietary compounds assembled from various commercial vendors (Enzo, Selleck, ChemBridge, Enamine, Life Sciences) for screening targets in HTS. Eight molecular properties were calculated for this collection using the Accelrys Pipeline Pilot application. An analysis showed that 89.7% of compounds have molecular properties matching all eight criteria for lead-like molecules to serve as starting points for a drug discovery effort (Molecular Weight ≤ 500; Heteroatom count ≤ 10; Number Rotatable Bonds ≤ 8; Number Aromatic Rings ≤ 4; A Log *p* ≤ 6; Molecular Polar Surface Area ≤ 200; H-bond acceptors < 10; H-bond donors < 5). Within this chemical space, the collection is diverse, containing: (1) 278,767 non-overlapping Murcko scaffolds with an average cluster size 2 to 3, (2) 9228 individual ring systems with unique substitution pattern (average frequency 218), and (3) 16,008 contiguous ring systems with unique substitution patterns (average cluster frequency 125). A subset of 674,336 compounds from this collection were tested in HTS format at a single concentration of 10 µg/mL or 30 µM depending on the compound library source.

Assay Method: Library compounds were diluted in assay medium (DMEM with 10% FBS, 1% PSG, 1% HEPES) to prepare a 3.5× concentrated dosing solution (35 µg/mL or 105 µM) and added to 384-well black clear bottom plates (Corning; Cat # 3764BC) in 10 µL (1/3.5 final well volume). Twenty-five µL of Producer cells (HEK 293 FT cells stably expressing GaLV Env and pNL4-3 ΔEnv-GFP) at 800,000 Cells/mL was added to the plates for a final count of 20,000 Cells/well, a final compound concentration of 10 µg/mL or 30 µM, and a final DMSO concentration of 0.5%. Assay medium alone (at 0.5% DMSO) served as the negative control (columns 1 and 2 of each plate) and 0.5 µM MLN4924 (Activebiochem; Cat # MLN4924) (at 0.5% DMSO) as the positive control (columns 23 and 24 of each plate). The plates were incubated at 37 °C/5% CO_2_ for 24 h in a humidified atmosphere. Ten µL of supernatant was transferred from each plate into a new 384-well black clear bottom plate, and 20 µL of Acceptor cells (293FT cells stably expressing mCAT-1 and the MLV Env receptor) at 300,000 Cells/mL added to the plates now containing 10 µL supernatant for a final count of 6000 Cells/well. The plates were returned to 37 °C/5% CO_2_ for 72 h and GFP signal imaged on a Mirrorball plate reader (BMG Labtech). The fluorescence signals of the test wells were normalized to percent activation relative to the average of the positive control wells on each plate by the formula:

% activation = 100 × (1 − (test well signal- average positive control signal)/(average negative control signal—average positive control signal))

Potential hits from this primary HTS were cherry-picked and tested at ten concentrations in a two-fold serial dilution over a concentration range of 50–0.1 µM or µg/mL, in both the main assay outlined above as well as in a counter screen assay.

HTS Counter Screen: Compounds were diluted in assay medium (DMEM with 10% FBS, 1% PSG, 1% HEPES) to prepare a 3.5×concentrated dosing solution and added to 384-well black clear bottom plates (Corning; Cat # 3764BC) in 10 µL (1/3.5 final well volume). Twenty-five µL of Acceptor cells (293FT cells stably expressing mCAT-1 and the MLV Env receptor) was added to the plates for a final count of 6000 Cells/well, a final compound concentration of 50–0.1 µM or 50–0.1 µg/mL, and a final DMSO concentration of 0.5%. Assay medium alone (at 0.5% DMSO) and 0.5 µM MLN4924 (at 0.5% DMSO) served as controls. The plates were incubated at 37 °C/5% CO_2_ for 24 h in a humidified atmosphere, after which GFP signal was imaged on a Mirrorball plate reader (BMG Labtech).

### 2.6. Cellular Target Counter Screens

293FT cells stably expressing CDC25α-GFP or APOBEC3G-GFP (described above) were treated with 40 μM compound, DMSO, or MLN4924 for 24 h at 37 °C and collected for flow cytometry. Mean Fluorescence Intensity (MFI) was measured.

### 2.7. CD4 and Tetherin Surface Labeling in PBMCs

PBMCs from HIV-negative donors were CD8-depleted (Invitrogen Cat# 11147D) and activated with 0.5 μg/mL purified PHA (Remel Cat# R30852801) in complete media (RPMI-1640 with glutamax (Gibco Cat# 61870-036) containing 10% FBS (Peak Serum Cat# PS-FB1), 1% HEPES (Gibco Cat# 15630-080), and 1% Pen/Strep (Gibco Cat# 15140-122)) supplemented with 20 IU/mL rhIL-2 (R&D Systems Cat# 202-IL) for 72 ± 4 h at 37 °C and 5% CO_2_. PHA was removed and washed out with complete media. 2.5 × 10^6^ P were resuspended in either 5 mL of VSV-g pseudotyped HIV-1_NL4-3ΔEnv-eGFPΔVpu_, HIV-1_NL4-3ΔEnv-eGFPΔNefΔVpu_ or HIV-1_NL4-3ΔEnv-eGFPΔNef_ viral supernatants or infection media control (DMEM with glutamax (Gibco Cat# 10566-016) with 10% FBS, and 1% MEM Non-Essential Amino Acids (Gibco Cat# 11140-050)), aliquoted into 24 well plates, and spinoculated at room temperature for 2 h at 2000 rpm with no brake applied. Following spinoculation, the viral supernatant was removed and replaced with 1 mL complete media with 20 IU/mL rhIL-2. After 24 ± 4 h, compounds were added to each well at the final concentration of 25 μM. Following additional incubation for 24 ± 4 h at 37 °C and 5% CO_2_, the supernatant was removed, and cells were treated with an antibody cocktail containing 1:400 Live/Dead stain (InVitrogen Cat# L34965), 1:100 CD4 stain (BioLegend Cat# 300530, clone RPA-T4), and 1:100 BST-2/Tetherin stain (BioLegend Cat# 348415, clone RS38E) in 3% FBS in PBS (Gibco Cat# 14190144) in the dark at 4 °C for 30 min. Cells were washed 3 times with 3% FBS in PBS, fixed in 2% PFA (Affymetrix Cat#199431LT) in PBS, and analyzed using flow cytometry on Stratedigm S1000Exi (Stratedigm, Inc., San Jose, CA, USA).

### 2.8. CD4 Surface Labeling in TZM-GFP

TZM-GFP cells were transduced with VSV-G pseudotyped packaged HIV-1 constructs containing either gag and env and lacking vif, vpr, pol and vpu or containing gag and env and lacking vif, vpr, pol and vpu. Both constructs contain mCHERRY in place of the Pol gene. The media was changed on day four post-infection, and cells were treated with 40 μM SRI-41897, SRI-42371, 1 μM MLN4924, or 0.1% DMSO for 24 h at 37 °C and 5% CO_2_. For surface staining, cells were lifted using TrypLE Express and transferred to round bottom 2 mL tubes in 1 mL of PBS. Cells were pelleted at 300 RCF for 3 min and the supernatant was removed. Cells were resuspended in a blocking solution containing 5% goat serum in PBS at 4 °C for 30 min. Cells were pelleted at 300 RCF for 3 min and the blocking solution was removed. Staining was done in a 1% goat serum and PBS solution containing 1:100 APC conjugate CD4 Stain (Life Technologies REF# MHCD0405) at 4 °C for 1 h in the dark. Cells were pelleted at 300 RCF for 3 min, and the stain was removed. Cells were washed 3 times in 1mL of PBS. Following washes, cells were fixed in 1% PFA in PBS. PFA solution was removed after centrifugation for 3 min at 300 RCF, and cells were resuspended in 400 μL of PBS and analyzed on n Accuri C6 flow cytometer.

### 2.9. Flow Cytometry

Cells were washed with PBS and lifted using Tryple Express (Gibco). Cells were removed from the plate and moved to 1.5 mL microcentrifuge tubes containing PBS and paraformaldehide (PFA) at a final concentration of 4% PFA for 10 min at room temperature. Next, cells were spun down at 800 RCF for 5 min and washed 2 times with PBS before the final resuspension in PBS and were analyzed using an Accuri C6 flow cytometer.

### 2.10. 2G12 Surface Labeling

Surface Labeling 2G12 was previously described [47]. Infected primary CD4 T cells were treated with compound 24 h post infection. 48 h post infection, cells were incubated for 20 min at 37 °C, with 5 μg/mL 2G12 (AB002; Polymun). Cells were then washed once with PBS and incubated with 1 μg/mL anti-human (Alexa Fluor 647; Invitrogen, Waltham, MA, USA) secondary Abs and the viability dye AquaVivid (Thermo Fisher Scientific, Waltham, MA, USA) for 15 min in PBS. Cells were washed again with PBS and fixed in a 2% PBS-formaldehyde solution. Infected cells were stained intracellularly for HIV-1 p24, using a Cytofix/Cytoperm fixation/permeabilization kit (BD Biosciences, Mississauga, ON, Canada) and fluorescent anti-p24 MAb (phycoerythrin [PE]-conjugated anti-p24, clone KC57; Beckman Coulter/Immunotech). The percentage of infected cells (p24^+^) was determined by gating the living cell population on the basis of viability dye staining (Aquavivid; Thermo Fisher Scientific). Samples were acquired on an LSR II cytometer (BD Biosciences), and data analysis was performed using FlowJo vX.0.7 (Tree Star, Ashland, OR, USA).

### 2.11. ADCC

The ADCC assay was previously described [45]. Target cells were infected 4 days prior to each assay with either WT NL4-3 HIV-1 or NL4-3 HIV-1 ∆Vpu. CEM.NKR-CCR5 cells were infected by spinoculation for 2 h at 1200 RCF. After spinoculation, the virus was removed, and target cells were cultured in R10 medium (RPMI medium supplemented with 10% FBS, L-glutamine, and Primocin). Immediately before the assembly of ADCC assays, the infected target cells were washed three times in R10 medium. 24 h after infection, compounds were added to the infected target cells at a concentration of 25 μM for each independently. Forty-eight h after infection, target cells were washed then incubated with the NK cell line KHYG-1 at a ratio of 10:1 of 150,000 effector cells to 15,000 target cells in round-bottom, tissue culture-treated polystyrene 96-well plates. Assays were performed in R10 culture medium containing 10 U IL-2 per mL, with no CsA. For controls, each plate contained NK effector cells and uninfected target cells in the absence of antibody (resulting in a 0% relative light units (RLU)), and NK cells and infected targets in the absence of antibody (resulting in 100% RLU). Serial, 4-fold, triplicate dilutions of plasma or monoclonal antibody were added. Each compound was once again used to treat the cell mixture at 25 μM. Once targets, effectors, and serially diluted antibody were combined, assay plates were incubated for 8 h at 37 °C and 5% CO_2_. After an 8 h incubation, a 150 μM volume of cells was resuspended and mixed by pipette with 50 μM of the luciferase substrate reagent BriteLite Plus (Perkin Elmer, Waltham, MA, USA) in white 96-well plates. Luciferase activity was read approximately 2 min later using a Wallac Victor3 plate reader (Perkin Elmer). 50% ADCC titers were estimated as previously described for virus neutralization assays [46]. The 50% intercept was calculated using the adjacent%RLU values above and below 50% RLU.

## 3. Results

### 3.1. High Throughput Screen for Vpu Inhibitors

**Figure 1:**
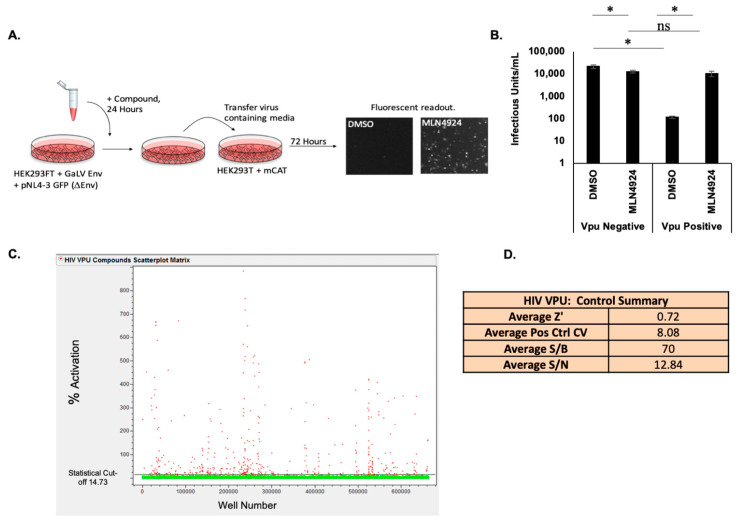
High Throughput Screen for Vpu Inhibitors (**A**) Schematic of GaLV Infectivity Assay experimental design. Cells stably expressing GaLV/MLV Env were transduced with a HIV-1 provirus. Cells were treated with compound for 24 h, and viral media was used to infect cells expressing the MLV receptor. Vpu blockage results in higher infectivity. (**B**) GaLV Infectivity Assay. Loss of infectious particle production is Vpu dependent and is rescued by MLN4924. Data is represented on a logarithmic scale. N = 3, and error bars represent standard deviation. ns = *p* value > 0.05, * = *p* value ≤ 0.05. (**C**) Scatter plot of HTS data. The threshold for selecting active compounds (red symbols) is 14.73% (line) as determined by the mean + 3 × SD of all the data. (**D**) Statistical values showing HTS assay performance determined from high (positive) and low (negative) control values on each plate. Z’ = 1 − 3(StdDevHiControl + StdDevLowControl)/(AvgHiControl—AvgLowControl); % Pos Cont CV = 100 × StdDevHiControl/AvgHiControl; S/B (Signal/Background) = Avg HiControl/AvgLowControl; S/N (Signal/Noise) = (AvgHiControl − AvgLowControl)/sqrt(StdDevHiControl^2^ + StdDevLowControl^2^).

Our lab previously showed that Vpu prevents infectious particle production in HIV-1 particles pseudotyped with a GaLV Env chimera consisting of a Murine Leukemia Virus (MLV) Envelope containing a GaLV Env c-terminal-domain (CTD) (henceforth referred to as GaLV Env) that is targeted by Vpu for downmodulation [38]. Using this observation, we developed a gain-of-function high throughput screen (HTS) for Vpu inhibitors (Figure 1A). This screen uses HEK 293 FT cells stably expressing GaLV Env and pNL4-3 ΔEnv-GFP to produce virus followed by the transduction into 293FT cells stably expressing mCAT-1, the MLV Env receptor, as target cells [39]. The cells produce HIV-1 viral particles pseudotyped with GaLV Env that successfully transduce mCAT-1 expressing cells in the absence of Vpu and produce few to no viral particles if the NL4-3 provirus contains a functioning Vpu (Figure 1B). The compound MLN4924 is a Neddylation inhibitor that effectively blocks the function of the cullin-Ring group of ubiquitin ligases and therefore blocks the cellular machinery that Vpu depends on for much of its activity [15,16,37,48]. In the absence of Vpu or in the presence of an inhibitor blocking Vpu, such as MLN4924, infectious particle production is rescued (Figure 1B).

This assay was used in the HTS to screen 674,336 commercially available compounds for Vpu inhibition (Figure 1C,D). The average Z’- value calculated from the 32 positive and 32 negative control wells on each plate was 0.72, with a range from 0.51 to 0.85 for the 2145 assay plates run in the screen. A Z’-value > 0.5 indicates that the assay performance was adequate to detect active compounds when tested once [49]. A statistical analysis (mean + 3 × SD of all test compound data) identified 14.73% inhibition as the cutoff between inactive and active compounds. Based on this cutoff, a total of 461 compounds were identified as hits resulting in an overall hit rate of 0.07% for the primary screen. Of the 461 hit compounds identified, 202 were confirmed as active following retest at ten concentrations in the GFP reporter assay used in HTS. These compounds were also evaluated for concentration-response in counter screen assays measuring compound autofluorescence or non-Vpu mediated activation of GFP expression. Based on these results, 177 compounds were excluded for causing autofluorescence or having off-target effects. The remaining 25 compounds were selected for further testing in secondary assays, of which 21 were available for purchase as fresh powders. Further information for hits that are not further explored in this manuscript is available in Supplemental Document S1.

### 3.2. HTS Reveals Potent Vpu Inhibitors

**Figure 2:**
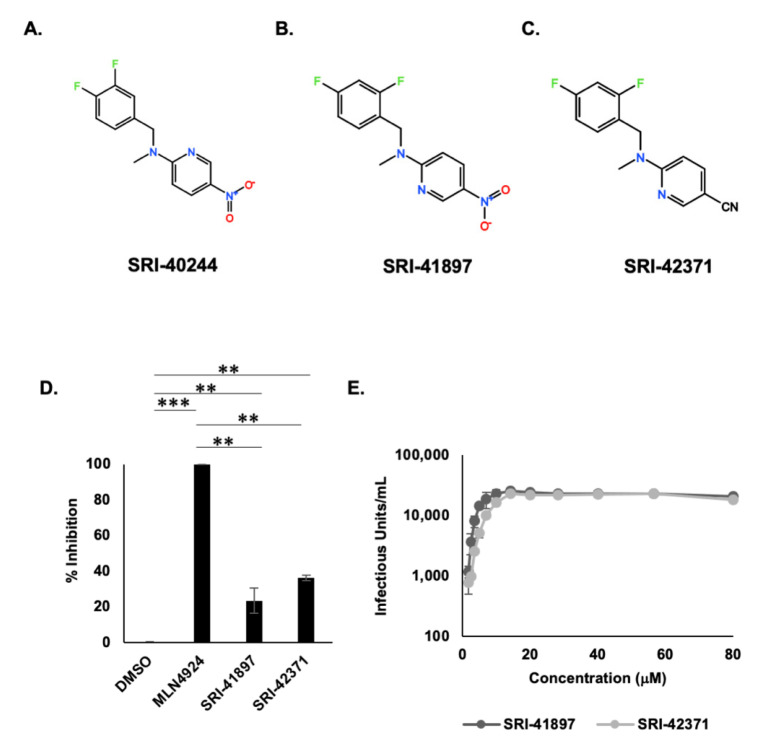
HTS reveals potent Vpu Inhibitors. (**A**) Structures of SRI-40244, (**B**) SRI-41897, (**C**) SRI-42371 drawn on TouchMol Structure drawing program available online. (**D**) GaLV infectivity assay rescue shown normalized to MLN4924. Compounds added at 40 μM in ‘semi-stable’ transduced cells as described in Figure 1A. Error bars reflect standard deviation, N = 4. ** = *p* value ≤ 0.01, *** = *p* value ≤ 0.001. (**E**) EC50 was determined using the GaLV infectivity assay (Figure 1A) for compound concentrations ranging from 0.88 to 80 μM. Error bars reflect standard deviation. N = 3. The EC50 was calculated using the AAT Bioquest EC50 calculator available online. The calculated EC50 for SRI-41897 and SRI-42371 were 4.4 μM and 7.4 μM, respectively.

One compound, SRI-41897, inhibited Vpu more potently than others. Interestingly, SRI-41897 had a very similar structure to another of the 21 HTS hits, SRI-40244, highlighting the potential for the structural family as Vpu inhibitors (Figure 2A,B). While multiple occurrences of similarly structured compounds are interesting, SRI-40244 had very low activity in the GaLV inhibition assay and was not pursued further (Supplemental Document S1). As we were interested in this structural class of compounds, we screened more than 80 additional commercially available analogs for SRI-41897. Most of the analogs contained the same background molecule with the addition, removal, or exchange of individual side chains. From the screened analogs, we found SRI-42371 that similarly inhibited Vpu in the GaLV Inhibition assay compared to the original compound (Figure 2D). These two compounds became the focus of our study. The EC_50_ of both compounds is in the μM range, 4.4 and 7.4 μM for SRI-41897 and SRI-42371, respectively, as measured in the GaLV inhibition assay (Figure 2E).

### 3.3. Vpu- Independent Counter Screens

**Figure 3:**
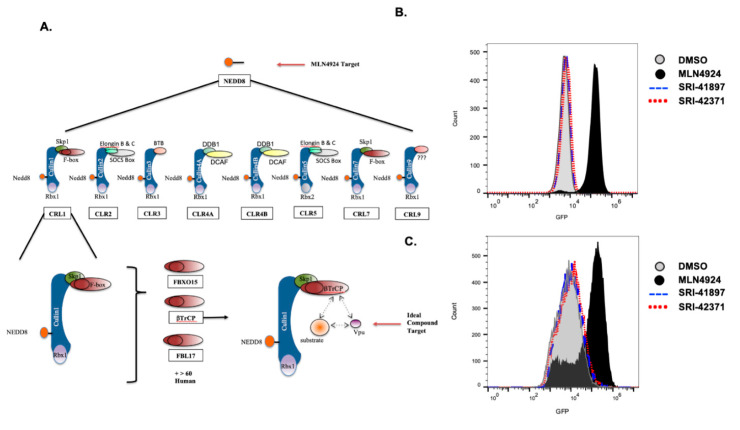
Vpu-Independent Counter Screens. (**A**) Schematic of potential upstream targets of Vpu inhibitors. Vpu depends on a specific cellular ubiquitin ligase (Cullin1) and a specific F-box protein (βTrCP). Blockage of any upstream cellular machinery could lead to a positive hit in the HTS or GaLV assay while not being specific to Vpu. (**B**) Cells expressing GFP-tagged APOBEC3G and HIV-1 Vif were treated were treated with either 40 μM of SRI-41897, SRI42371 or one of the controls, DMSO or MLN4924 for 24 h and collected for flow cytometry. Mean fluorescence intensity (MFI) is shown in representative images from a total of four biological replicates. (**C**) Cells expressing GFP-tagged CDC25α were treated with either 40 μM of SRI-41897, SRI42371 or one of the controls, DMSO or MLN4924 for 24 h and collected for flow cytometry. Mean fluorescence intensity (MFI) is shown in representative images of four total biological replicates.

While the HTS is Vpu dependent, compounds that block the cellular machinery that Vpu depends on will also result in a positive hit. To ensure that the compounds are specific for Vpu, we developed a series of counter screens for the upstream cellular machinery maintaining MLN4924 as a positive control (Figure 3A).

The first counter screen ensures that the compounds do not have any activity for the broader cullin family of ubiquitin ligases. In this assay, 293FT cells that are stably expressing both GFP tagged APOBEC3G and the HIV-1 accessory protein, Vif, were treated with compounds, either DMSO or MLN4924, and MFI was recorded via flow cytometry (Figure 3B). Vif has previously been shown to downmodulate the cellular protein APOBEC3G using cullin5 machinery [50,51,52,53], so a blockage of cullin5 results in an increase of APOBEC3G expression and therefore an increase in GFP as shown by MFI. Addition of MLN4924 showed a clear increase in MFI of GFP, whereas neither SRI-41897 nor SRI-42371 displayed an increase in fluorescence, indicating that they do not block cullin5 or the larger cullin ubiquitin ligase family.

The second counter screen ensures that the compounds do not have any activity against the F-box protein, βTrCP 1 or 2. In this assay, 293FT cells stably expressing GFP tagged CDC25α were treated with the compounds DMSO or MLN4924 as a positive control, and MFI was recorded via flow cytometry (Figure 3C). βTrCP has been previously shown to downmodulate the cellular protein CDC25α; therefore, a blockage of βTrCP results in an increase of CDC25α expression and an increase in GFP as shown by MFI [54,55]. The addition of MLN4924 resulted in a clear increase in fluorescence; however, neither SRI-41897 nor SRI-42371 displayed an increase in fluorescence, indicating that they do not block βTrCP function.

### 3.4. Compounds Rescue CD4 and BST-2/Tetherin Expression

**Figure 4:**
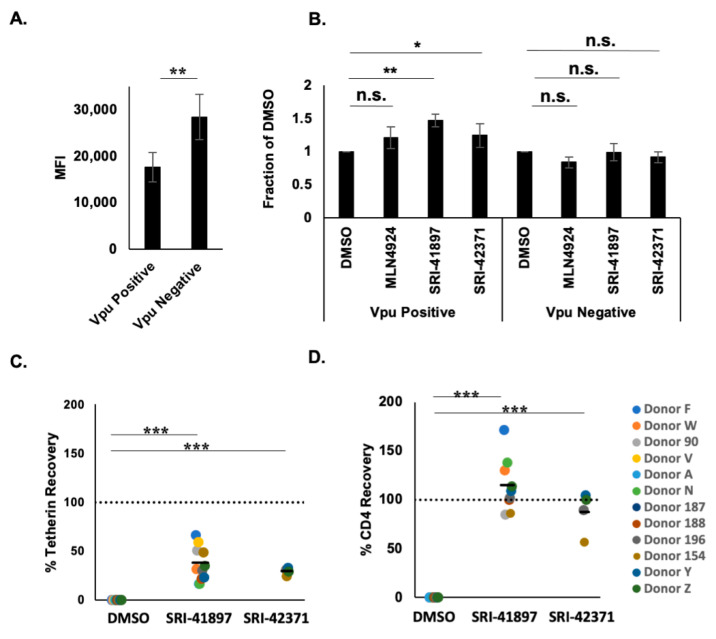
Compounds Rescue CD4 and Tetherin Expression. (**A**) Surface Expression of CD4 in the presence and absence of Vpu. N = 4. TZM-GFP cells were transduced with VSV-G pseudotyped HIV-1 constructs containing mCHERRY. Day 5 post-infection, cells were surface stained for CD4 and analyzed by flow cytometry. Error bars represent standard deviation. ** = *p* value ≤ 0.01. (**B**) Surface Expression of CD4 in the presence and absence of Vpu after treatment with compounds. N = 4. Cells were surface labeled as in (**A**), with the addition of 40 μM of SRI-41897 or SRI-42371 on day 4. Post-infection data is normalized to DMSO. Error bars represent standard deviation. N = 4. ns = *p* value > 0.05, * = *p* value ≤ 0.05, ** = *p* value ≤ 0.01 (**C**) Tetherin surface labeling, and (**D**) CD4 surface labeling were done as follows: PBMCs from HIV-negative donors were CD8-depleted and activated. Cells were resuspended in VSV-g pseudotyped with HIV-1_NL4-3ΔEnv-eGFPΔNef_ viral supernatant. After 24 ± 4 h, compounds were added to each well at the final concentration of 25 μM for 24 h. Following this, the supernatant was removed and cells were surface labeled with an antibody cocktail containing Live/Dead stain, CD4 stain, and BST-2/Tetherin stain. Data was analyzed using flow cytometry. *** = *p* value ≤ 0.001.

To explore the impact of compounds on surface expression of CD4, TZM-GFP cells were transduced with a Gag-mRFP construct containing a non-functional Pol protein and a functional Env in the presence or absence of Vpu (Figure 4A,B). CD4 levels of Vpu positive cells had a MFI average of 1764.8, which is 61% of Vpu negative cells that had a MFI average of 28,472.5 (Figure 4A). After treatment with MLN4924 or compounds, CD4 surface expression was rescued by 47% or 25% on infected TZM-GFP cells, for SRI-41897 or SRI-42371, respectively, in comparison to the negative control, DMSO (Figure 4B). While the compounds had no effect on CD4 surface expression in the absence of Vpu, it is interesting that MLN4924 reduced CD4 surface expression in these cells. This is likely why the rescue of CD4 surface expression with MLN4924 in the presence of Vpu was not significant.

To determine if SRI-41897 and SRI-42371 can rescue the expression of CD4 and Tetherin in human peripheral blood mononuclear cells (PBMCs), cells were infected with HIV-1_NL4-3_, treated with compound for 24 h, and surface labeled. Surface levels of CD4 and BST-2/Tetherin were measured via flow cytometry. Tetherin expression was restored an average of 38% and 29% and CD4 expression was restored an average of 115% and 88% when treated with 25 μM SRI-41897 and SRI-42371, respectively, in PHA-activated, CD8-depleted PBMCs (Figure 4C,D).

### 3.5. Compounds Rescue ADCC Response

**Figure 5:**
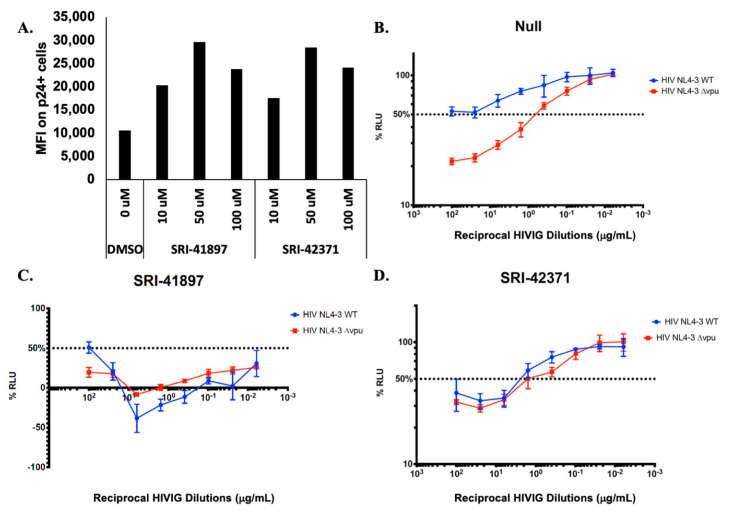
(**A**) NL4-3 Infected primary CD4 T cells were treated with compound 24 h post infection. After 48 h post infection, cells were surface stained with the 2G12 antibody. Additionally, infected cells were stained intracellularly for HIV-1 p24. Samples were analyzed using flow cytometry The percentage of infected cells (p24^+^) was determined by gating the living cell population on the basis of viability dye staining. N = 1. (**B**–**D**) N = 3. Compounds were added to infected target cells at a concentration of 25 μM each, independently. After 48-hours post infection, target cells were incubated with NK cells. For controls, each plate contained NK effector cells and uninfected target cells in the absence of antibody (0% relative light units (RLU)), and NK cells and infected targets in the absence of antibody (100% RLU). Serial, 4-fold, triplicate dilutions of plasma or monoclonal antibody were added. A luciferase signal was detected in each well. The 50% intercept was calculated using the adjacent %RLU values above and below 50% RLU. Error bars represent standard deviation.

Antibody Dependent Cellular Toxicity (ADCC) is a cellular response to infection that results in cell killing through lysis. In recent years, several groups have shown that Vpu protects HIV-1 infected cells from killing by ADCC, and that this effect is mostly due to downmodulation of CD4 and BST-2/Tetherin (reviewed in [24,56,57]). We wanted to test if the compounds could rescue ADCC as they rescued the expression of CD4 and partial expression of BST-2/Tetherin (Figure 4). Before testing ADCC, we examined Env expression using a conformationally-independent anti-HIV-1 Env antibody 2G12 expression [58,59]. Primary CD4^+^ T cells were infected with WT NL4-3 virus, treated with compounds at various concentrations, and probed with the anti-HIV-1 Env antibody, 2G12 (Figure 5A). We observed a dose dependent response in 2G12 binding, indicating that in the presence of compound there is more Env present on the surface of the cell. Next, target cells derived from CEM.NKR-CCR5_CD4+_ T cells expressing a Tat-inducible luciferase reporter gene were infected with either WT NL4-3 HIV-1 or NL4-3 HIV-1 ∆Vpu, co-cultured with natural killer (NK) cells and reciprocal anti-HIV-1 Immune Globulin (HIVIG) dilutions [45] (Figure 5B–D). In this assay, an increase in ADCC killing is shown through a decrease in luciferase signal (decrease in actively infected cells). In the absence of compound, ADCC levels are significantly higher in NL4-3 ∆Vpu infected cells when compared with NL4-3 WT infected cells (Figure 5B); however, the introduction of compound brought ADCC levels against NL4-3 WT ADCC close to ∆Vpu levels (Figure 5C,D), indicating that the presence of compounds rescue ADCC killing in NL4-3 WT infected cells.

### 3.6. Compounds Are NL4-3 Strain Specific

**Figure 6:**
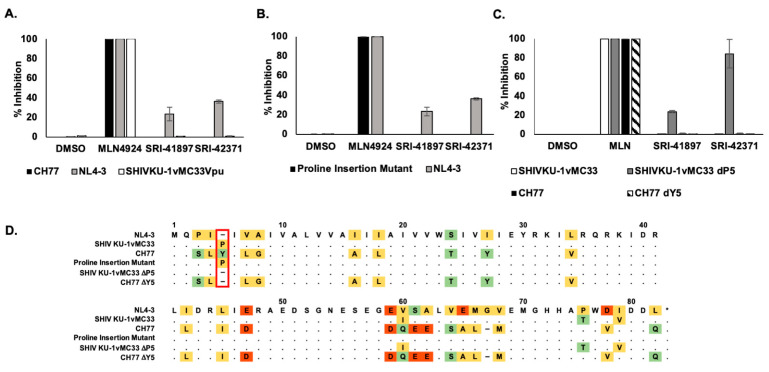
Compounds are NL4-3 Strain Specific. (**A**) N = 3. GaLV Infectivity Assay (Figure 1A) with NL4-3, CH77, and SHIV_KU-1vMC33_Vpu strains cloned into a NL4-3 backbone and normalized to MLN4924. Error bars represent standard deviation. (**B**) N = 3. GaLV Infectivity Assay (Figure 1A) comparison of NL4-3 Vpu and proline insertion mutant normalized to MLN4924 positive control. Error bars represent standard deviation. (**C**) N = 3. GaLV Infectivity Assay (Figure 1A) comparison of SHIV_KU-1vMC33_ and CH77 Vpu with two deletion mutants where the fifth amino acid is removed, all in a NL4-3 backbone normalized to an MLN4924 positive control. Error bars represent standard deviation. (**D**) Schematic of amino acid sequences of NL4-3, CH77, SHIV_KU-1vMC33_, Proline Insertion Mutant, and Deletion Mutant Vpu strains. Highlighting reflects discrepancies in relation to NL4-3 Vpu.

To test the breadth of Vpu strains that are inhibited by SRI-41897 and SRI-42371, we tested inhibition against a wide variety of available Vpu strains and discovered a strain very similar to NL4-3 Vpu that was resistant to both compounds; the strain is from a simian-human immunodeficiency virus strain, SHIV_KU-1vMC33_, containing a Vpu originally from HIV-1 HXB2 [60,61]. SHIV_KU-1vMC33_ Vpu contains only four amino acid changes from NL4-3 Vpu (Figure 6A,C). Additionally, another more clinically relevant strain of Vpu, CH77, was cloned into a NL4-3 backbone. While approximately 74% identical, there are several differences between CH77 and NL4-3 Vpu (Figure 6D). The NL4-3, CH77, and SHIV_KU-1vMC33_ strains of Vpu were examined in the GaLV inhibition assay (introduced in Figure 1A) in the presence and absence of compound or MLN4924 (Figure 6A). Interestingly, both compounds had no inhibition of CH77 Vpu or SHIV_KU-1vMC33_, but the positive control, MLN4924, still functioned to inhibit both Vpu strains.

The SHIV_KU-1vMC33_ Vpu and CH77 Vpu sequences differ in the same place from NL4-3 Vpu in only two places, AA5 and AA61 (Figure 6D). To examine the effect of this position on SRI-41897 and SRI-42371, a single proline, the same amino acid in position 5 of SHIV_KU-1vMC33_ was inserted into NL4-3 Vpu after amino acid 4 and tested alongside NL4-3 WT Vpu in the GaLV inhibition assay (Figure 6B). The single proline insertion was sufficient to completely abrogate the function of both compounds against Vpu. We also deleted the 5th amino acid from both SHIV_KU-1vMC33_ Vpu and CH77 Vpu, a proline and tyrosine, respectively, to determine if this deletion could rescue the effects of compounds on these two Vpu strains (Figure 6C). While deletion of Y5 in CH77 Vpu did not rescue compounds, deletion of P5 in SHIV_KU-1vMC33_ Vpu rescued SRI-41897 to similar levels as NL4-3 and rescue effects of SRI-41897 to a higher level than seen with NL4-3 Vpu. According to the Los Alamos database, less than 1% of Vpu contain single amino acid deletion between two side-by-side isoleucine residues at the N terminus like with NL4-3 Vpu. This suggests that the compounds would not likely be functional on most strains of Vpu.

## 4. Discussion

While still not fully elucidated, the inability to completely clear an HIV-1 infection could largely be due to accessory proteins, such as Vpu, that aid in immune system evasion through mechanisms such as the downmodulation of CD4, BST-2/Tetherin, and inhibitory effects on ADCC. Currently, there are no approved therapies targeting HIV-1 Vpu.

Here we described two small molecules, SRI-41897 and SRI-42371, identified from an HTS, that inhibit the HIV-1_NL4-3_ Vpu protein. The HTS took advantage of our previous finding that Vpu targets the cytoplasmic tail of GaLV Env using the same SCF/βTrCP ubiquitin ligase machinery it uses to downmodulate other targets [16,38,39]. This HTS has the advantage of being a gain-of-function assay reducing the chance of false-negative hits. Additionally, in a gain-of-function cellular screen, compounds must be able to cross the cell membrane to result in a positive hit, and highly toxic compounds will result in a negative signal.

Of the 21 HTS hits, two compounds were structurally similar, and one of them, SRI-41897, had a strong signal. Over 80 commercially available analogs of SRI-41897 were screened in the GaLV inhibition assay, and a second compound, SRI-42371 more potently inhibited Vpu (Figure 2). While SRI-42371 inhibited Vpu more potently than SRI-41897 in the GaLV inhibition assay, the EC_50_ of SRI-42371 was higher. Furthermore, the nitro group present on SRI-41897 raises a concern of potential toxicity (Reviewed in [62]). Thus, SRI-42371 may be considered a better hit for optimization.

Vpu specificity has been a limitation in production of Vpu inhibitors, so it was important to us to test potential off-site effects of SRI-41897 and SRI42371. To do this, compounds of interest were counter screened in a Vpu-independent manner for auto fluorescence (data not shown) and inhibition of the SCF/βTrCP ubiquitin ligase machinery that Vpu depends on at two different levels, broader cullin-family inhibition and the inhibition of cellular βTrCP 1 and 2 (Figure 3B,C). Both SRI-41897 and SRI-42371 had a negative signal for auto-fluorescence, cullin-family inhibition, and βTrCP inhibition, suggesting that they are specific to Vpu. This is a strength of SRI-41497 and SRI-42371 in comparison to the compound BIT225, which has been shown to increase plasma derived activated CD4^+^ and CD8^+^ T cells and NK cells if coupled with ART [34], and is not Vpu specific [33,35].

Next, we showed that SRI-41897 and SRI-42371 rescue CD4 and partial BST-2/Tetherin expression in PBMCs infected with HIV-1_NL4-3_ in a Vpu-dependent manner (Figure 4A,B). This result suggests that these two compounds hinder the functional activity of Vpu, which is an important factor in evasion of the immune system during a HIV-1 infection and is another limitation of the compound BIT225 [36]. Furthermore, in recent years, Vpu has been shown to be an important factor in protecting infected cells from killing by ADCC (reviewed in [25]). Excitingly, we also showed that SRI-41897 and SRI-42371 increase the surface Env expression and killing of infected cells by ADCC (Figure 5). This finding is important, as the clinical trial of the HIV-1 vaccine ALVAC/AIDSVAX (RV144), despite being only about 30% effective, did not impact viral load or CD4+ cell counts in vaccinated individuals who became infected [63]; however, upon further analysis, it was reported that ADCC may have contributed to the ~30% protection reported in the trial because of a reduced risk of infection in vaccinated individuals that had lower IgA titers [64]. The significance of heightened ADCC levels in HIV-1 infection control is also supported by higher ADCC levels reported in elite controllers not on ART [65,66], and by higher ADCC levels being correlated to a lower transmission rate through breastfeeding [67]. For this reason, for a Vpu inhibitor to be successful in a clinical setting, it would likely be necessary for the inhibitor to rescue the ADCC response. It is important to note that part of the effect of Vpu on ADCC protection is linked to its ability to downregulate BST-2/Tetherin [27,28,29]; however, the ability of Vpu to downregulate CD4^+^ also had a significant effect on ADCC susceptibility [26,68]. This further highlights the importance of a Vpu inhibitor that can rescue at least partial expression of both BST-2/Tetherin and CD4 if it were to be used in a clinical setting.

The positive hit in the HTS, the negative hit in both counter screens, and some CD4, BST-2/Tetherin, and ADCC rescue all suggest a dependence on Vpu. Unfortunately, we discovered that SRI-41897 and SRI-42371, specific to the NL4-3 strain of Vpu are presumed to interact primarily with the N-terminus of Vpu around proline 4 and 5 (Figure 6). The addition of a proline after isoleucine 4 abrogated the effect of SRI-41897 and SRI-42371. While disappointing, the additional data showing NL4-3 specificity and dependance on a specific amino-acid sequence strongly suggest that both compounds are directly interacting with Vpu towards the N-terminus rather than any other cellular or HIV-1 targets. The finding that SRI-41897 and SRI-42371 interact with the N-terminus before or right at the transmembrane region is interesting because much of the activity of Vpu has been attributed to the cytoplasmic tail, specifically Serine 52/56. However, the transmembrane region downstream of where we hypothesize compounds are interacting, specifically A14/18, has been shown to be important for complete BST-2/Tetherin downmodulation, but this is not the case for CD4 downmodulation [16,17,18,19]. An NMR structure of Vpu has been previously solved, and it is interesting to note that the group that changes in the transmembrane region of Vpu altered the angle of the cytoplasmic tail region [69]. Such alterations could alter the overall structure of Vpu and alter membrane trafficking and/or co-factor interactions. While further studies would be necessary, this is a possible explanation as to why a compound interacting at the N terminus of Vpu may disrupt functions associated with the cytoplasmic tail as changes in the cytoplasmic tail angle could impact binding of the ubiquitin ligase complex.

Even though SRI-41897 and SRI-42371 are NL4-3 specific, these two compounds have the potential to help further the understanding of Vpu and the effect its inhibition has on HIV-1 infection in both cell culture and animal models. Most importantly, we demonstrate here a solid proof-of-concept for high throughput screening for Vpu inhibitors. We were able to pull several small-molecule candidates from a large library of compounds, counter screen candidate compounds, demonstrate functional inhibition, and locate important residues for these compounds to interact with Vpu. This gain-of-function assay may easily be adapted to screen inhibitors for other, more clinically relevant, strains of Vpu.

## Supplementary Materials

The following supporting information can be downloaded at: https://www.mdpi.com/article/10.3390/v14040817/s1, Excel Document S1: AB Table Excel Tab: Contains the Supplier, Supplier ID number, and, when applicable, the assigned SRI number. 10K Excel Tab: Contains additional information on the first 10K compound screen hits including activity information. 660K Excel Tab: Contains additional information on compound activity, off-target activity, autofluorescence, and CD4 levels.
